# Impact of an Early Invasive Strategy versus Conservative Strategy for Unstable Angina and Non-ST Elevation Acute Coronary Syndrome in Patients with Chronic Kidney Disease: A Systematic Review

**DOI:** 10.1371/journal.pone.0153478

**Published:** 2016-05-19

**Authors:** Catriona Shaw, Dorothea Nitsch, Jasmine Lee, Damian Fogarty, Claire C. Sharpe

**Affiliations:** 1 UK Renal Registry, Southmead Hospital, Bristol, London, United Kingdom; 2 Department of Renal Medicine, Kings College London, London, United Kingdom; 3 London School of Hygiene and Tropical Medicine, London, United Kingdom; 4 Department of Renal Medicine, Guy’s and St Thomas’ NHS Foundation Trust, London, United Kingdom; 5 Department of Renal Medicine, Belfast Health and Social Care Trust, Belfast, Northern Ireland, United Kingdom; University of Bologna, ITALY

## Abstract

**Background:**

Clinical practice guidelines support an early invasive approach after NSTE-ACS in patients with chronic kidney disease (CKD). There is no direct randomised controlled trial evidence in the CKD population, and whether the benefit of an early invasive approach is maintained across the spectrum of severity of CKD remains controversial.

**Methods:**

We conducted a systematic review to evaluate the association between an early invasive approach and all-cause mortality in patients with CKD. We searched MEDLINE and EMBASE (1990-May 2015) and article reference lists. Data describing study design, participants, invasive management strategies, renal function, all-cause mortality and risk of bias were extracted.

**Results:**

3,861 potentially relevant studies were identified. Ten studies, representing data on 147,908 individuals with NSTE-ACS met the inclusion criteria. Qualitative heterogeneity in the definitions of early invasive approach, comparison groups and renal dysfunction existed. Meta-analysis of the RCT derived and observational data were generally supportive of an early invasive approach in CKD (RR0.76 (95% CI 0.49–1.17) and RR0.50 (95%CI 0.42–0.59) respectively). Meta-analysis of the observational studies demonstrated a large degree of heterogeneity (I^2^ 79%) driven in part by study size and heterogeneity across various kidney function levels.

**Conclusions:**

The observational data support that an early invasive approach after NSTE-ACS confers a survival benefit in those with early-moderate CKD. Local opportunities for quality improvement should be sought. Those with severe CKD and the dialysis population are high risk and under-studied. Novel and inclusive approaches for CKD and dialysis patients in cardiovascular clinical trials are needed.

## Introduction

In non ST elevation acute coronary syndrome (NSTE-ACS) an early invasive approach (coronary angiography followed by revascularisation if appropriate) is the recommended strategy in patients with hemodynamic instability, refractory angina, electrical instability, or an elevated risk for clinical events [1]. Although patients with chronic kidney disease (CKD) are recognised as high risk for poor outcomes after NSTE-ACS, analyses of real world cohorts have consistently suggested that they are less likely to undergo angiography or revascularisation than those with normal kidney function [2–4]. The reasons behind these practice patterns are complex. Studies have reported under-estimation of risk, despite paradoxically high GRACE scores [3]. Additionally concerns regarding risk of acute kidney injury (AKI) or a perceived excess bleeding risk are likely to contribute. Practice patterns may also reflect underlying uncertainty as to whether the overall survival benefit conferred by an early invasive strategy compared with a selective strategy is maintained across the spectrum of CKD.

There is no direct randomised controlled trial (RCT) evidence available and CKD patients have historically being under-represented in the relevant clinical trials [5]. A prior meta-analysis of from five major cardiac trials (n = 1,453) that had collected renal data reported that an early invasive strategy was associated with non-significant reductions in all-cause mortality in those with CKD stage 3–5 (eGFR <60ml/minute/1.73m^2^) [6]. However, whether the results are generalizable to contemporary non trial CKD patients, in particular those with severe CKD, is not known. The overall prevalence of CKD was relatively low at 19% compared with 30–40% reported in registry based analyses [4, 7] and despite pooling data from several clinical trials, the power of the analysis was limited. 267 participants had CKD stage 4/5 and no data was available on outcomes in dialysis patients. The most recent trial included was published in 2005 and in the decade since management of both CKD and ACS have evolved. Although the meta-analysis is supportive of an early, routine invasive approach in CKD patients with NSTE-ACS the evidence is not definitive.

In the absence of direct RCT data, several observational studies using real world NSTE-ACS populations have been published. No rigorous systematic review exists to evaluate the quality of the evidence and summarise the additional data they provide on this important clinical question.

This review sought to assess systematically the published observational evidence to evaluate whether an early invasive strategy compared with an initial conservative strategy after NSTE-ACS is associated with an improvement in survival in patients across the spectrum of CKD.

## Subjects and Methods

### Types of Studies/Participants

Studies comparing a routine early invasive strategy with a conservative strategy after NSTE-ACS or unstable angina (UA) in adults (aged>18 years) with CKD.

### Types of Interventions

The management strategies compared were:

1. An early invasive approach with coronary angiography +/- revascularisation if appropriate
2. An initial conservative approach where coronary angiography +/- revascularisation was not routinely undertaken and patients were managed with a non-invasive strategy initially

We anticipated there may be variation in the definitions used within studies. To provide an inclusive approach in this review we did not specify further the definitions of early invasive approach compared with initial conservative strategy.

#### Outcome

All-cause mortality.

### Search Methods

MEDLINE (1990-19^th^ May 2015) and EMBASE (1990-19^th^ May 2015) were searched without language restrictions or study type restrictions (S1 Appendix). Reference lists were hand-searched of relevant review articles identified and of the studies included in this systematic review.

### Study Selection

CJS reviewed all the articles identified via the literature search for potential inclusion. The selection process was undertaken in three stages- first, by reviewing all the identified article titles; secondly abstracts of relevant articles were reviewed, and finally the full text versions of selected articles were reviewed. A 10% sample of the publications at each stage was checked by a second independent reviewer (JBL). Concordance was >80%.

### Data Extraction

CJS extracted data from the included studies using pre-designed, piloted extraction forms.

### Assessment of Risk of Bias

CJS assessed risk of bias using the Cochrane Collaboration tool for cohort studies. Domains evaluated included: selection of participants, comparability of study groups and the ascertainment of outcomes of interest and overall risk of bias (S2 Appendix).

For the RCTs within the prior systematic review [6], risk of bias was evaluated in terms of blinding to outcome assessment, intention to treat analysis for the primary analysis and losses to follow-up.

### Data Synthesis and Statistical Analysis

A narrative synthesis is provided. Summary statistics for the included studies are provided. For studies that reported an effect estimate and standard error or confidence intervals results were meta-analysed, stratified by whether the study was an RCT or observational. Meta-regression was undertaken to explore the effect of study size on heterogeneity between the studies. All analyses were conducted using STATA v12. This study was reported using PRSIMA guidelines (S3 Appendix).

## Results

**Fig 1:**
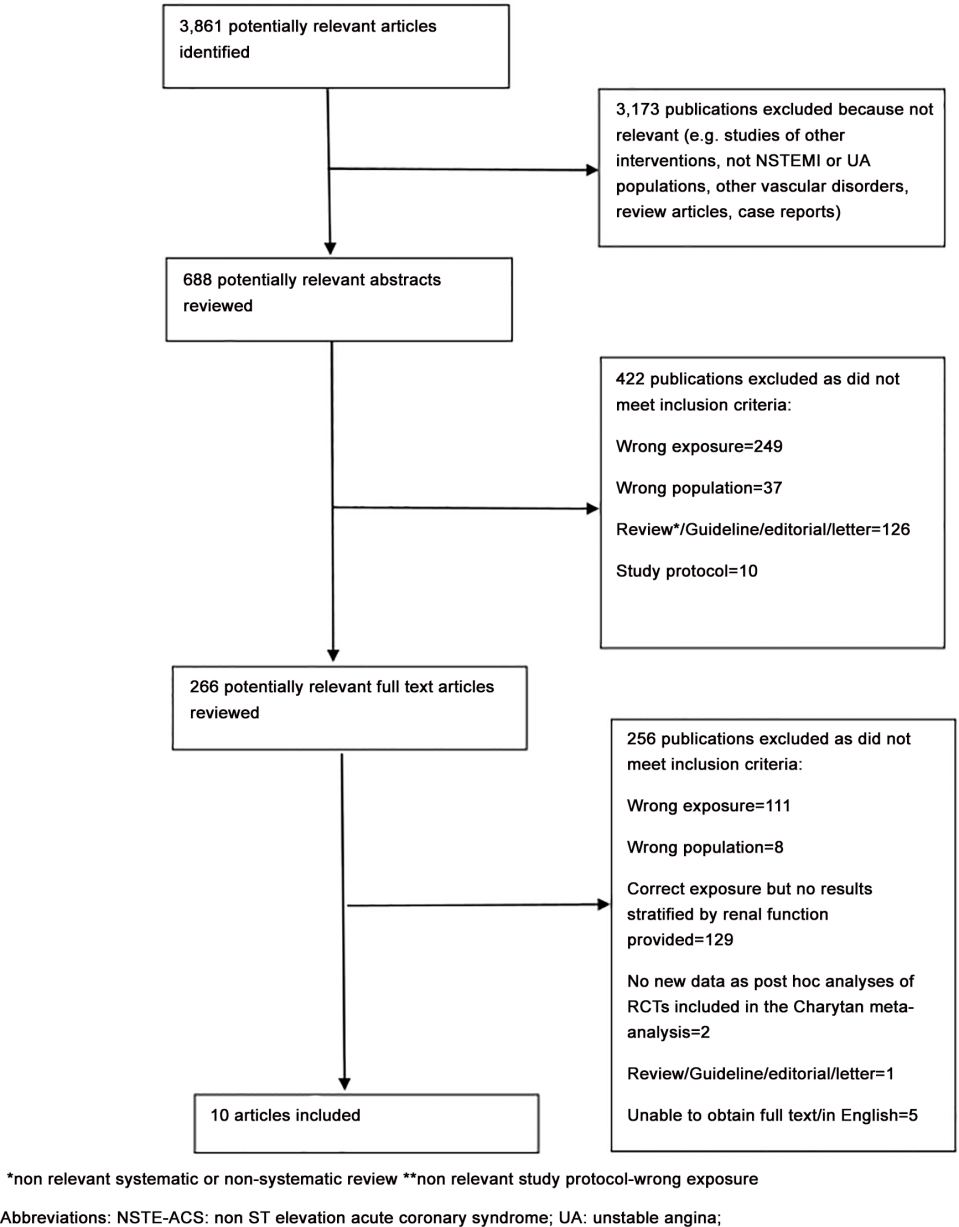
Flow chart showing exclusion process during the literature review. *non relevant systematic or non-systematic review **non relevant study protocol-wrong exposure. Abbreviations: NSTE-ACS: non ST elevation acute coronary syndrome; UA: unstable angina.

The search strategy identified 3,861 unique citations (Fig 1). Ten studies met the inclusion criteria and were included in the review [3, 4, 6, 8–14] (Tables 1 and 2).

**Table 1:** Summary of the studies included in the systematic review.

First author (country) Year of publication	Data source/ Patient Population (years of study)	Exposure and comparison group definitions	Definition of kidney disease (type of creatinine based estimating equation)	Study design and Risk of Bias	Outcome and main result
Altahan et al (Israel)2011	ACSIS registry/ NSTE-ACS (2004–2008)	In patients with and without kidney disease 3 groups were compared:1)Early in-hospital coronary angiography (<48 h from admission) 2) Late in-hospital coronary angiography (>48 h from admission)3) No angiography performed	eGFR<60ml/minute (eGFR:4 point MDRD)	Retrospective cohort design using registry data; MEDIUM risk of bias	**Primary Outcome**: 1 yr all-cause mortality **Result: Early angiography compared with no angiography as IP**: eGFR>60ml/minute: adj HR 0.34(0.18–0.65) eGFR<60ml/minute: adj HR 0.46(0.31–0.68) **Late angiography compared with no angiography as IP:** eGFR>60ml/minute: adj HR 0.58(0.34–1.01); eGFR<60ml/minute: adj HR 0.59(0.43–0.82)
Bhatt et al (USA)2004	CRUSADE registry/ NSTE-ACS (2000–2002)	In patients with and without kidney disease 2 groups were compared: 1)Cardiac catheterization within 48 hours of hospital presentation 2)No cardiac catheterization within 48 hours of hospital presentation	Creatinine>176.8micromol/L Crcl<30ml/minute or chronic renal dialysis (Cockcroft and Gault))	Retrospective cohort design using registry data linked to a quality improvement programme; MEDIUM risk of bias	**Primary Outcome**: in hosp all-cause mortality **Result: Cardiac catheterization within 48 hours compared with no cardiac catheterization within first 48 hours:** No kidney disease: adj OR 0.68(0.55–0.88); Kidney disease: adj OR 0.58(0.37–0.90)
Chertow et al (USA) 2004	CCP/NSTE-ACS (1994–1995)	In patients with kidney disease who were judged appropriate for in-patient angiography 2 groups were compared **: 1)in patient angiography (+/ revascularisation) 2)no inpatient angiography(+/- revascularisation)	Creatinine ≥1.5mg/dl (132micromol/L) and <5mg/dl (442micromol/L) Patients with creatinine >5mg/dl or on dialysis were excluded	Retrospective cohort design using data from an observational study of treatment patterns and outcomes of acute MI in the elderly; MEDIUM risk of bias	**Primary Outcome**: 1 yr all-cause mortality **Result: In patient coronary angiography compared with no in patient coronary angiography in patients with kidney disease**: adj OR 0.54(0.49–60)
Charytan et al (FRISC II-Scandinavia; TIMI IIIB US; TACTICS TIMI 18 international; ICTUS-Dutch VINO-Czech republic) 2011	TIMI IIIB, FRISC II, TACTICS-TIMI 18, VINO, ICTUS (1989–2003)	In patients with CKD stage 3–5 2 groups were compared 1)early invasive strategy 2)conservative strategy	eGFR<60ml/minute (eGFR: 4 point MDRD)	Individual level meta-analysis of RCT data; LOW risk of bias	**Primary Outcome:** all-cause mortality at 6months-1 year **Result: early invasive strategy compared with conservative strategy in those with CKD3-5:** adj RR 0.77 (0.49–1.17)
Chu et al (Taiwan)	NHIRD (2005–2008)	In patients with and without kidney disease 2 groups were compared: 1)diagnostic coronary angiography (with intent to revascularise) within 72 hours of symptom onset 2)no diagnostic coronary angiography within 72 hours of symptom onset	CKD defined using ICD-9 codes 585.4, 585.5, 585.9 without dialysis or ARF(ICD9 code 584) 6 months prior to admission	Retrospective cohort design using data from single payer National Health Insurance program (covers 99.9% of Taiwanese population): MEDIUM/HIGH risk of bias	**Primary Outcome:** Major adverse cardiac events (cardiovascular death, MI and stroke) **Result:** Kaplan Meier survival curves plotted. MACE free survival was lowest in those with kidney disease undergoing EIS
Goldenberg et al(Israel, Europe) 2010	EUPHORIC project/ NSTE-ACS and UA (2000, 2004, 2005, 2006)	In patients with and without kidney disease 2 groups were compared: 1)inpatient coronary angiography (+/-revascularisation) 2)no inpatient angiography(+/- revascularisation)	eGFR<60ml/minute (eGFR:4 point MDRD)	Retrospective cohort design using registry data (from 3 registries ACSIS,MASCARA and EHS-ACS: MEDIUM risk of bias	**Primary Outcome**: all-cause mortality–in hospital **Result: Inpatient coronary angiography compared with no inpatient coronary angiography**: eGFR≥60ml/minute: adj OR 0.45(0.26–0.78); eGFR<60ml/minute: adj OR 0.64(0.41–1.00); P-interaction (eGFR) = 0.31
James et al (Canada) 2013	APPROACH/AKDN NSTE-ACS and UA (2004–2009)	Outcomes stratified by renal function at time of presentation with NSTE-ACS were presented comparing 2 groups: 1) coronary angiography within 2 days of admission with NSTE-ACS (+/- revascularisation) within 2)no coronary angiography within first 2 days	Patients were categorised by first eGFR at admission (within first 2 days) eGFR>/ = 60/minute eGFR 30-59/minute eGFR<30/minute (eGFR: CKD EPI)	Retrospective cohort design using linked registry data (ACS with provincial administrative healthcare and laboratory data): MEDIUM/LOW risk of bias	**Primary Outcome:** All-cause mortality (Dec 2009) **Result: Coronary angiography within 2 days compared with no coronary angiography within 2 days:** eGFR> = 60/minute = adj HR 0.74(0.56–0.99); eGFR 30-59/minute = adj HR 0.66(0.52–0.85); eGFR<30/minute = adj HR 0.55(0.31–0.91); (overall = adj HR 0.69 (0.58–0.82); p-interaction 0.624
Lin et al (Taiwan) 2014	Taiwan ACS full spectrum registry (2008–2010)	Outcomes In patients with renal presentation at time of presentation with NSTE-ACS were presented comparing 2 groups: 1)diagnostic angiography within 72hours of symptom onset (with intent to revascularise) 2)no diagnostic angiography within 72 hours of symptom onset	Patients were categorised by renal function at time of admission eGFR>60ml/minute eGFR<60ml/minute (eGFR: CKD-EPI)	Retrospective cohort design using registry data: MEDIUM risk of bias	**Primary Outcome:** 1 yr mortality (composite of death, non-fatal MI and non-fatal stroke) **Result: Diagnostic angiography within 72 hours compared with no diagnostic angiography within 72 hours:** eGFR<60ml/minute: adj HR 0.53(0.30–0.92)
Shaw et al (UK) 2014	MINAP/NSTE-ACS (2008–2010)	Outcomes stratified by renal function at time of presentation with NSTE-ACS were presented comparing 2 groups: 1)in hospital coronary angiography(+/-revascularisation) 2)no in-hospital coronary angiography (or revascularisation)	Patients were categorised by renal function at time of admission: eGFR>90 ml/minute, eGFR 60–90 ml/minute, eGFR 30–60 ml/minute; eGFR <30 ml/minute (eGFR: CKD-EPI)	Retrospective cohort design using registry data; MEDIUM risk of bias	**Primary Outcome**: all-cause mortality at 1 year **Result: In-hospital angiography compared with no in-hospital coronary angiography:** eGFR 45-59ml/minute adj OR 0.37(0.32–0.43); eGFR 30-44ml/minute adj OR 0.41(0.34–0.48); eGFR<30ml/minute adj OR 0.46(0.36–0.58); p-interaction <0.001
Wong et al (Canada) 2009	ACS1 and ACS2 (1997–2007)	In patients with kidney disease 2 groups were compared 1)in hospital coronary angiography(+/-revascularisation) 2)no in-hospital coronary angiography (or revascularisation)	Patients were categorised by renal function: eGFR≥60ml/minute; eGFR30-59ml/minute; eGFR<30ml/minute including dialysis; (eGFR:4 point MDRD)	Retrospective cohort design using registry data; MEDIUM/HIGH risk of bias	**Primary Outcome**: 1 yr all-cause mortality **Result: In-hospital angiography compared with no in-hospital coronary angiography:** unadjusted 1 year mortality rates were lower among patients receiving in-hospital coronary angiography for all levels of kidney dysfunction

Abbreviations: eGFR: estimated glomerular filtration rate (ml/minute/1.73m^2^); adj HR: adjusted hazard ratio; adj OR: adjusted odds ratio; adj RR-adjusted risk ratio; MDRD:Modified diet in Renal Disease; CKDEPI:Chronic Kidney Disease Epidemiology Collaboration

ACSIS: Acute Coronary Syndromes Israeli Survey; CRUSADE: Can Rapid Risk Stratification of Unstable Angina Patients Suppress Adverse Outcomes With Early Implementation of the ACC/AHA Guidelines; CCP: Cooperative Cardiovascular Project; FRISC II Fragmin and Fast Revascularization during Instability in Coronary Artery Disease; VINO: Value of first day angiography/ angioplasty In evolving Non-ST segment elevation myocardial infarction; TIMI IIIB: Thrombolysis in Myocardial Infarction (TIMI) IIIB clinical trial; TACTICS TIMI18: The Treat Angina with Aggrastat and determine Cost of Therapy with an Invasive or Conservative Strategy; ICTUS: Invasive versus Conservative Treatment in Unstable Coronary Syndromes; NIHRD National Health Insurance Research Database; EUPHORIC: European Public Health Outcome Research and Indicators Collection Project; APPROACH: Alberta Provincial Project for Outcome Assessment in Coronary Heart Disease; AKDN: Alberta Kidney Disease Network; MINAP: Myocardial Ischaemia National Audit Project; ACSI/II: Canadian Acute Coronary Syndromes I and II

* estimated from Fig within published paper

** 92 clinical indicators were used to categorise individuals to an “appropriateness” score for angiography

**Table 2:** Characteristics of the randomised controlled trials included in the systematic review by Charytan *et al* [6].

	VINO [17]	FRISC II [15]	TIMI IIIB [16]	TACTICS-TIMI 18 [18]	ICTUS [19]
Full study title (Year of publication)	Value of first day angiography/ angioplasty In evolving Non-ST segment elevation myocardial infarction(2002)	Fragmin and Fast Revascularization during Instability in Coronary Artery Disease (2001)	Thrombolysis in Myocardial Infarction (TIMI) IIIB clinical trial (1995)	The Treat Angina with Aggrastat and determine Cost of Therapy with an Invasive or Conservative Strategy (2001)	Invasive versus Conservative Treatment in Unstable Coronary Syndromes(2005)
Interventions	First day angiography *vs*. symptom/stress test-driven angiography	First week angiography *vs*. symptom/stress test driven angiography; Dalteparin vs. placebo	Angiography within 18–48 hours *vs*. symptom/stress test-driven angiography; TPA vs. placebo	Angiography within 4–48 hours *vs*. symptoms/stress test driven angiography	Angiography within 24–48 hours *vs*. symptom /stress test-driven angiography
Type of ACS	Non-STEMI	Non-STEMI and UA	Non-Q wave MI and UA	Non-STEMI and UA	Non-STEMI
Renal exclusions	NA	Creatinine >1.8mg/dl (>159micromol/L)	Creatinine >3mg/dl (>265micromol/L)	Creatinine >2.5mg/dl (>221micromol/L)	NA
Primary end point (time point)	Composite of death and nonfatal MI (6months)	Composite of death and nonfatal MI (6 months)	Composite of death and nonfatal MI or “unsatisfactory” stress test (6 weeks)	Composite of death and nonfatal MI and hospitalisation for acute coronary syndrome (6 months)	Composite of death and nonfatal MI or hospitalisation for angina (1 year)

Abbreviations: TPA: tissue plasminogen activator; non-STEMI: non ST elevation myocardial infarction; UA: unstable angina; MI: myocardial infarction; CABG: coronary artery bypass graft; PTCA: percutaneous transluminal coronary angioplasty; CHF: congestive heart failure; NA: not applicable

Nine of the included studies were retrospective cohort studies using data from national or regional level registries (Table 1) [3, 4, 8–14]. The remaining study was the individual level meta-analysis of five RCTs conducted by Charytan *et al* [6].

147,908 individuals presenting between 1989–2010 with NSTE-ACS were included across the ten studies (Tables 3 and 4). The mean age of individuals in the cohort studies ranged from 63–75 years (Table 3). In the RCTs the mean age of the recruited population was 63 years, although in those with CKD it was higher (68 years) [6]. Cohort size varied from 57,284 individuals (CCP) [10] to 834 people (NHIRD) [11]. 60% (n = 87,432) of included participants were male (9 studies with data available). The prevalence of CKD varied between 10–40%. Where data on eGFR<30ml/minute/1.73m^2^ was reported the prevalence ranged from 0.9% (n = 4) in the FRISC II trial [6, 15] to 9% (n = 3,238) within the MINAP cohort study [4] (Tables 3 and 4). The prevalence of diabetes varied between 8% (TIMI III) [6, 16]and 41% (NHIRD) [11](Tables 3 and 4).

**Table 3:** Selected demographic and clinical characteristics of the cohort based study populations.

Main author (data source)	Altahan (ACSIS)	Bhatt (CRUSADE)	Chertow (CCP)	Chu (NHIRD)	Goldenberg (EUPHORIC)	James et al (APPROACH/ ADKN) ^1^	Lin (Taiwan ACS Full Spectrum) ^2^	Shaw (MINAP)	Wong (ACSI, ACS II)
No. in study	1,960	17,926	57,284	834	13,141	6,774	1,462	35,881	5,165
Age, year (mean)	68	68^a^	Age categories	64	66^b^	64	63	75^a^	70
Men (n,[%])	1,517(76)	10,573(59)	29,789(52)	517(62)	8,943(68)	4,805(71)	Data not provided for NSTE-ACS group	22,425(63)	3,760(73)
White ethnicity (n,[%])	Not provided	13,967(78)	51,987(91)	Not applicable	Not provided	Not provided	Not applicable	Not provided	Not provided
Renal dysfunction (n, [%])	eGFR<60 = 720(36)	Cr>178 or Cr Cl<30 or on dialysis = 2,475(14)	Creat >1.5mg/dl & <5mg/dl = 15,093(26)	By ICD9 codes = 82(10)	eGFR>/ = 60 = 8,960(68) eGFR<30–59 = 3,439(26) eGFR<30 = 742(6)	eGFR>/ = 60 = 3,898(58) eGFR30-59 = 2,728(40) eGFR<30 = 148 (2)	eGFR<60ml = 1,226(40)	eGFR>90 = 6,482(18) eGFR60-90 = 13,719 (38) eGFR 30–60 = 12, 442 (35) eGFR15-29 = 2,665 (7.4) eGFR<15 = 573 (1)	eGFR>/ = 60 = 3,294(64) eGFR30-59 = 1,599(31) eGFR<30 = 272 (5)
Diabetes (n, [%])	782(39)	5,870(33)	18,323(32)	342(41)	3,787(29)	1,546(23)	1,212(39)	8,560(24)	1,405(27)
Previous MI (n,[%])	699(35)	5,607(31)	16,734(29)	Not provided	Not provided	1,482(22)	(Previous CAD) 744(24)	11,976(33)	1,888(37)
EIS (n,[%])	337/1,960(17)	8,037(45)	23,540(41)	466(56)	8,151(62)	3384(50)	Not provided for the NSTE-ACS population	16,646(46)	2,504(48)
EIS by renal function^c^ (n,[%])	eGFR>/ = 60 = 718(57); eGFR<60 = 337(48)	Cr<178micromol/L = 7,507(49); Cr>178micromol/L or Cr Cl<30 or on dialysis = 530 (21)	Creat<1.5mg/dl = 19,735 (47); Creat >1.5mg/dl & <5mg/dl = 3,805 (25)	No KD = 433(56) KD = 33 (40)	eGFR>/ = 60 = 6,065(68); eGFR<60 = 2,086 (50)	eGFR>/ = 60 = 1,949 (58); eGFR30-59 = 1,364 (40) eGFR<30 = 74(2)	Not provided for the NSTE-ACS population	eGFR>90 = 4,720 (28); eGFR 60–90 = 7,445(54) eGFR 45–59 = 2,613(37) eGFR 30–44 = 1,366(25) eGFR<30 = 502 (16)	eGFR>/ = 60 = 1,794(54) eGFR30-59 = 638 (40) eGFR<30 = 72 (26)

Abbreviations: MI: myocardial infarction; EIS: early invasive strategy; PCI: percutaneous coronary intervention; CABG: coronary artery bypass graft; KD: kidney disease (as defined within the study); Creat: creatinine; CrCl: creatinine clearance; eGFR: estimated glomerular filtration rate

^1^ data presented is that in the propensity matched cohort n = 6,774; prior to propensity matching the cohort consisted of 10,516 adults who met the cohort criteria

^2^ this analysis presented composite baseline data for patients with ST elevation ACS and NSTE-ACS (N = 3,093) stratified by eGFR category only. No descriptive data was provided on demographics and clinical characteristics for the NSTE-ACS population only other than the number of patients = 1,462. Where data was available for the NSTE-ACS group only it is provided in the Table.

^a^ median

^b^ data averaged across data sources

^c^ percentage presented of those managed with EIS

**Table 4:** Selected demographic and clinical characteristics of the RCT based populations included in the meta-analysis by Charytan *et al [6].*

	**TIMI IIIB N = 1,473**	**FRISC II N = 2,457**	**TACTICS-TIMI 18 N = 2,220**	**VINO N = 131**	**ICTUS N = 1,200**
Age, year (mean)	59	66^a^	62	66	62 ^a^
Men (n,[%])	972(66)	1,708(70)	1,463(66)	80(61)	880(73)
White ethnicity (n,[%])	1178(80)	NA	1722(78)	NA	NA
Diabetes (n, [%])	114(8)	299(12)	613(28)	33(25)	166(14)
Renal dysfunction (n, [%])	eGFR<60 = 449(30)	eGFR<60 = 429(17)	eGFR<60 = 429(19)	eGFR<60 = 29(22)	eGFR<60 = 117(10)
**Characteristic in those with eGFR<60ml/minute/1.73m**^**2**^					
	**N = 449**	**N = 429**	**N = 429**	**N = 29**	**N = 117**
White ethnicity (n, [%])	403(90)	429(100)	340(79)	29(100)	NA
eGFR <30 (n, [%])	216 (48)	4(0.9)	29(7)	10(3)	8(7)
Diabetes (n, [%])	39(9)	75(17)	146 (34)	18 (62)	29 (25)
Previous MI (n, ([%])	188 (42)	146 (34)	188 (44)	15(52)	43(37)
ST-segment changes (n,[%]):	176(39)	237(55)	170 (40)	20 (69)	52 (44)
EIS/ECS (n, [%])	221/228 (49/51)	211/218(49/51)	216/213(50/50)	12/17(41/59)	58/59(50/50)
Coronary revascularisation during follow-up (invasive/conservative strategies) (n, [%])	147/144 (66/63)	158/101 (74/46)	123/90 (57/42)	6/7 (50/41)	38/27 (66/45)

EIS: early invasive strategy; ECS: early conservative strategy; eGFR: estimated glomerular filtration rate (ml/minute/1.73m^2^); MI:myocardial infarction

^a^ median

Qualitatively there was variation between the studies. There were a range of definitions used for early invasive strategy (TableS 1 and 2). The RCTs included in the meta-analysis all utilised similar definitions (Table 2). The most variation was seen in the FRISC II trial defining early invasive strategy as within the first week [15], compared to within the first 24–48 hours (Table 2) [6, 16–19]. The cohort studies used three variants of definition (Table 1). The CRUSADE and APPROACH analyses used a time frame of initial coronary angiography within the first 48 hours, compared with those that did not undergo coronary angiography in that time frame [9, 13]. The ACSIS study used the same early intervention definition but compared outcomes with those who did not undergo angiography during the index admission [8]. The NHIRD and Taiwan ACS Full Spectrum studies defined early invasive strategy as within 72 hours [11, 14]. The CCP, EUPHORIC, MINAP and the ACSI/II based studies compared individuals who underwent inpatient coronary angiography with those that did not (Table 1) [3, 4, 10, 12].

The definition of CKD varied. All studies defined renal disease using biochemical parameters except the NHIRD (ICD-9 codes on financial claim forms) [11] (Table 1). The CCP, APPROACH and NHIRD based analyses excluded dialysis patients [10, 11, 13]. In contrast, ACSI/II and CRUSADE based studies specifically commented that dialysis patients were included [3, 9]. In ACSI/II they were incorporated into eGFR<30/minute/1.73m^2^ (N = 639) and in the CRUSADE study in the binary definition of renal insufficiency used. Several trials included in the RCT meta-analysis had exclusion criteria equating to moderate to severe CKD (Table 2)[6]. No data were provided regarding patients with renal transplants.

The trials in the RCT meta-analysis were judged to be low risk of bias [6]. No cohort studies were judged to be of overall low risk of bias (Table 1 and S2 Appendix). Several studies reported exclusions to reduce risk of immortal time bias [4, 13]. Processes of validation and quality assurance were variably reported resulting in several studies being judged at risk of information bias. The use of single renal function measures across all the studies introduced the risk of CKD misclassification.

All studies presented multivariable analyses except the NHRID and ACSI/II analyses were univariable [3, 11]. Common variables adjusted for included age, sex, diabetes and previous coronary artery disease. Most confounder variables were in binary/categorical format introducing risk of residual confounding. Potentially important confounders such as ethnicity, socioeconomic status or adjunctive medical therapies were not routinely included (S4 Appendix).

### Study Specific Results

**Fig 2:**
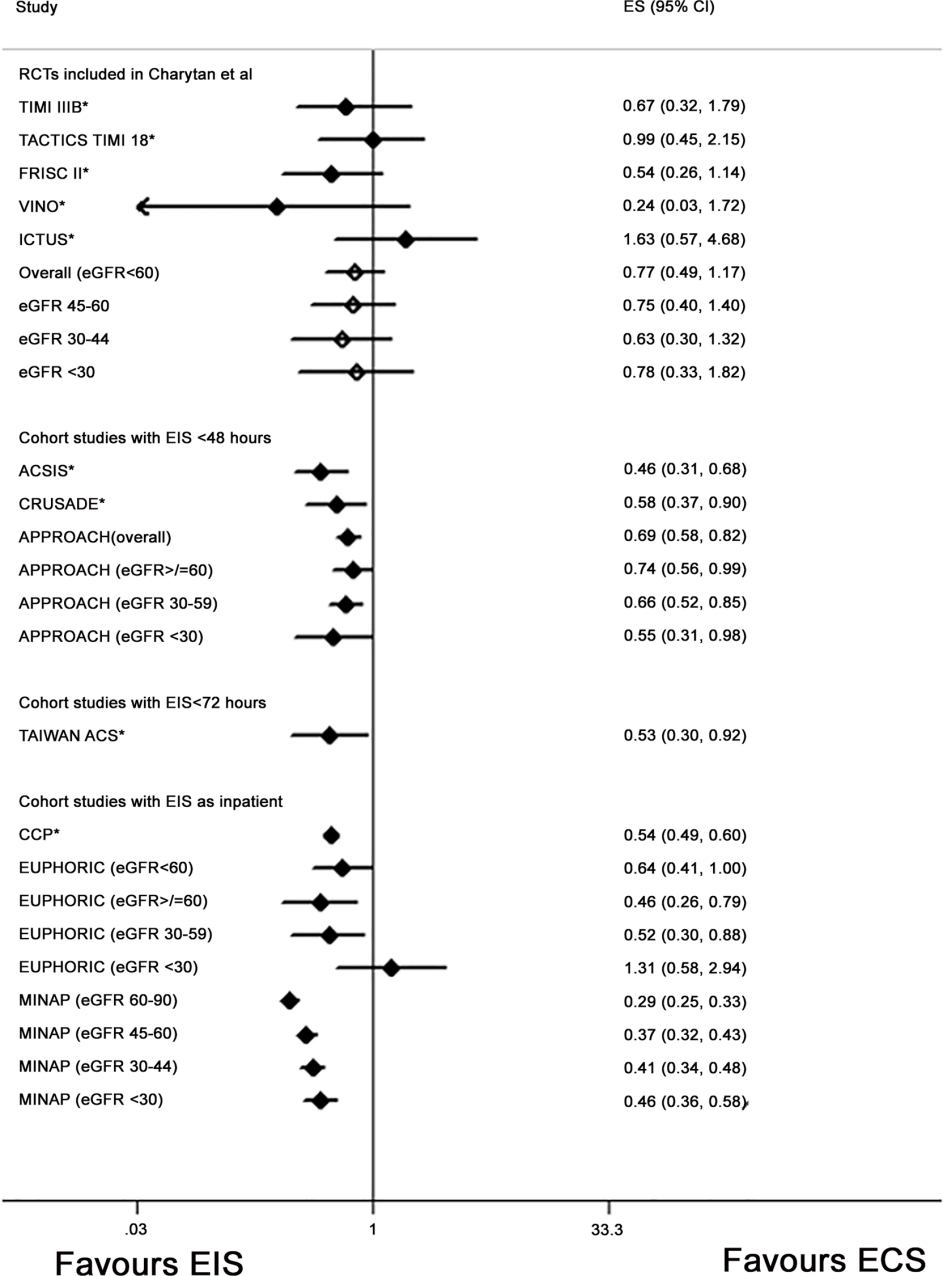
Forest plot illustrating study specific effects for the association between an early invasive strategy and mortality compared with a conservative management strategy with results stratified by estimated glomerular filtration rate. *the effect estimate presented is for those with an eGFR<60ml/minute/1.73m^2^ or the definition of kidney disease used within the specific study. For these studies results stratified further into subcategories of eGFR/CKD stages were not available. Results from the random effects meta-analysis conducted by Charytan *et al* are reported. The overall effect estimate for those with an eGFR<60ml/minute/1.73m^2^ is reported and then also results of the analysis further stratified by eGFR category (hollow diamonds). Results from the APPROACH study were reported overall for eGFR<60ml/minute/1.73m^2^ and also stratified by eGFR category (p-interaction by eGFR 0.624). Results from the EUPHORIC study were reported overall for eGFR<60ml/minute/1.73m^2^ and also stratified by eGFR category (p-interaction by eGFR 0.31). Results from the MINAP study were reported stratified by eGFR category (p-interaction by eGFR <0.001). Effect estimates for each of the studies included in the plot: ACSIS[8]- adjusted hazard ratio; CRUSADE[9]-adjusted odds ratio; APPROACH [13]-adjusted risk ratio; Charytan *et al* [6]- risk ratio; Tai (Taiwan ACS full spectrum registry) [14]-adjusted hazard ratio; CCP[10]- adjusted odds ratio; EUPHORIC [12]- adjusted odds ratio MINAP [4]–adjusted odds ratio. Abbreviations: EIS: early invasive strategy; ECS: early conservative strategy; ES: effect estimate; 95% CI: 95% confidence interval; TAIWAN ACS: TAIWAN ACS Full Spectrum Registry; ACSIS: Acute Coronary Syndromes Israeli Survey; CRUSADE: Can Rapid Risk Stratification of Unstable Angina Patients Suppress Adverse Outcomes With Early Implementation of the ACC/AHA Guidelines; CCP: Cooperative Cardiovascular Project; FRISC II Fragmin and Fast Revascularization during Instability in Coronary Artery Disease; VINO: Value of first day angiography/ angioplasty In evolving Non-ST segment elevation myocardial infarction; TIMI IIIB: Thrombolysis in Myocardial Infarction (TIMI) IIIB clinical trial; TACTICS TIMI18: The Treat Angina with Aggrastat and determine Cost of Therapy with an Invasive or Conservative Strategy; ICTUS: Invasive versus Conservative Treatment in Unstable Coronary Syndromes; EUPHORIC: European Public Health Outcome Research and Indicators Collection Project; APPROACH: Alberta Provincial Project for Outcome Assessment in Coronary Heart Disease; MINAP: Myocardial Ischaemia National Audit Project.

All the cohort studies that reported multivariable adjusted results reported a survival benefit associated with an early invasive strategy compared to a conservative strategy in those with CKD (Table 1 and Fig 2) [4, 8–10, 12–14]. Relative benefits of between approximately 30–50% were reported across the cohort studies (Table 1 and Fig 2). The NHIRD reported unadjusted Kaplan Meier survival probabilities which suggested that an early invasive strategy in those with CKD was associated with a poorer survival probability than conservative strategy [11] (Table 1). This was in contrast to the univariable results presented by the ACSI/II analyses [3] (Table 1).

Where outcomes were stratified by CKD stage there was a suggestion that the survival benefit may be attenuated in those with CKD 4/5- except in the APPROACH study (eGFR<30: adj HR 0.55 (95% CI 0.31–0.98)[13] (Fig 2). In two studies however the confidence intervals crossed one, likely reflecting the low numbers of patients within this CKD class in those studies (EUPHORIC eGFR<30: adj OR 1.31(95% CI 0.58–2.94); Charytan *et al* eGFR<30: RR 0.78(0.33–1.82) [6, 12]. The APPROACH and EUPHORIC studies reported no evidence of interaction by eGFR on the association between early invasive strategy and mortality in contrast to the MINAP report (Table 1 and Fig 2)[4].

**Fig 3:**
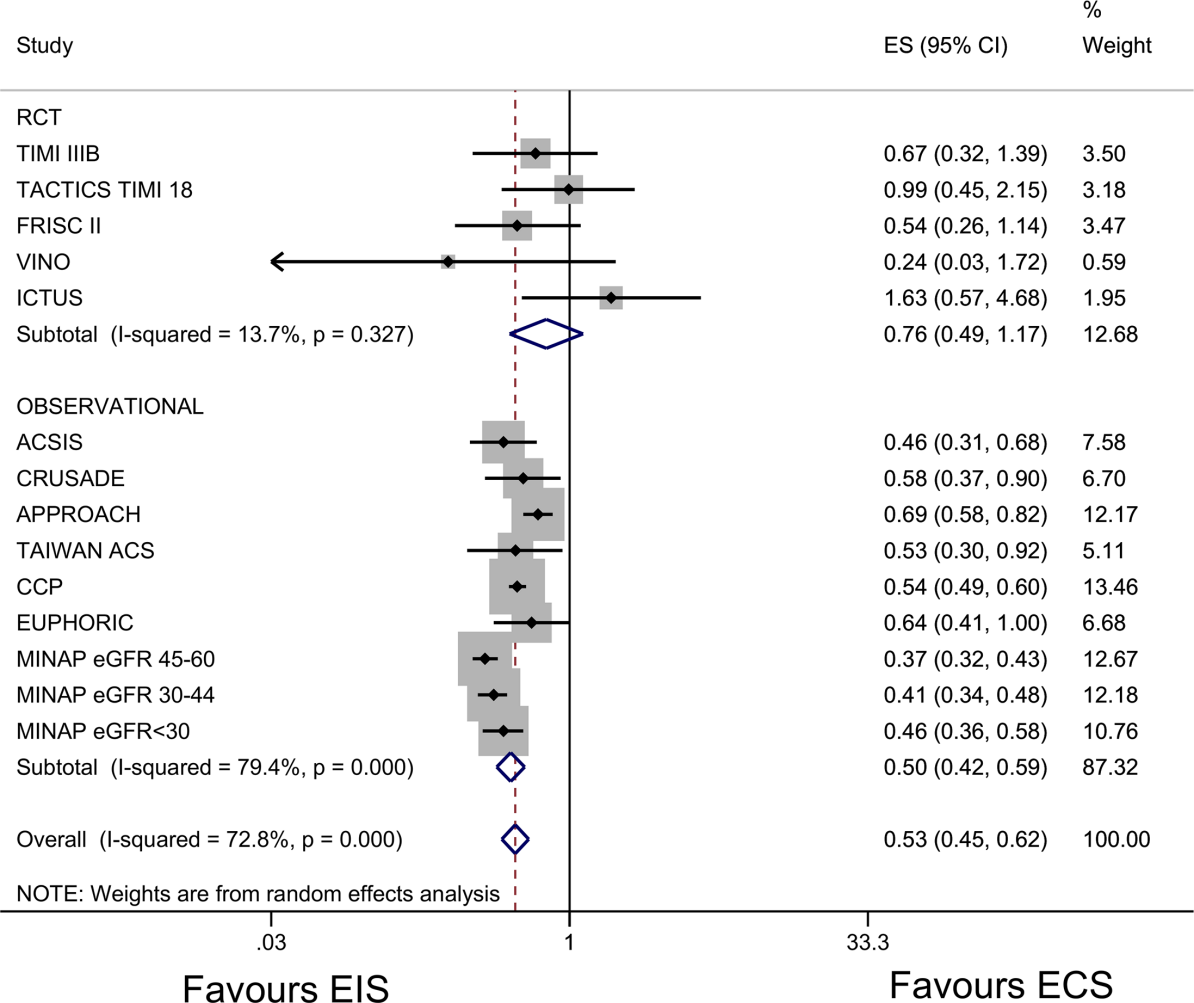
Forest plot and meta-analysis across all stages of kidney function for the association between an early invasive strategy and mortality compared with a conservative management strategy with results stratified by estimated glomerular filtration rate. Effect estimates for each of the studies included in the plot: ACSIS[8]- adjusted hazard ratio; CRUSADE[9]-adjusted odds ratio; APPROACH [13]-adjusted risk ratio; Charytan *et al* [6]- risk ratio; Tai (Taiwan ACS full spectrum registry) [14]-adjusted hazard ratio; CCP[10]- adjusted odds ratio; EUPHORIC [12]- adjusted odds ratio MINAP [4]–adjusted odds ratio. Abbreviations: EIS: early invasive strategy; ECS: early conservative strategy; ES: effect estimate; 95% CI: 95% confidence interval; TAIWAN ACS: TAIWAN ACS Full Spectrum Registry; ACSIS: Acute Coronary Syndromes Israeli Survey; CRUSADE: Can Rapid Risk Stratification of Unstable Angina Patients Suppress Adverse Outcomes With Early Implementation of the ACC/AHA Guidelines; CCP: Cooperative Cardiovascular Project; FRISC II Fragmin and Fast Revascularization during Instability in Coronary Artery Disease; VINO: Value of first day angiography/ angioplasty In evolving Non-ST segment elevation myocardial infarction; TIMI IIIB: Thrombolysis in Myocardial Infarction (TIMI) IIIB clinical trial; TACTICS TIMI18: The Treat Angina with Aggrastat and determine Cost of Therapy with an Invasive or Conservative Strategy; ICTUS: Invasive versus Conservative Treatment in Unstable Coronary Syndromes; EUPHORIC: European Public Health Outcome Research and Indicators Collection Project; APPROACH: Alberta Provincial Project for Outcome Assessment in Coronary Heart Disease; MINAP: Myocardial Ischaemia National Audit Project.

Meta-analysis of the RCT derived data across all CKD stages suggested a trend to improved survival although the 95% confidence interval crossed one (adjusted HR 0.77 (95% CI 0.49–1.17)(Fig 3) with an I^2^statistic of 14%. Meta-analysis of the observational studies also supported an early invasive strategy however there was marked between study heterogeneity (I^2^ statistic 79%). After meta-regression for study size this reduced to 64%. Removal of the MINAP study from the meta-analysis reduced the I^2^ statistic to 30%.

**Fig 4:**
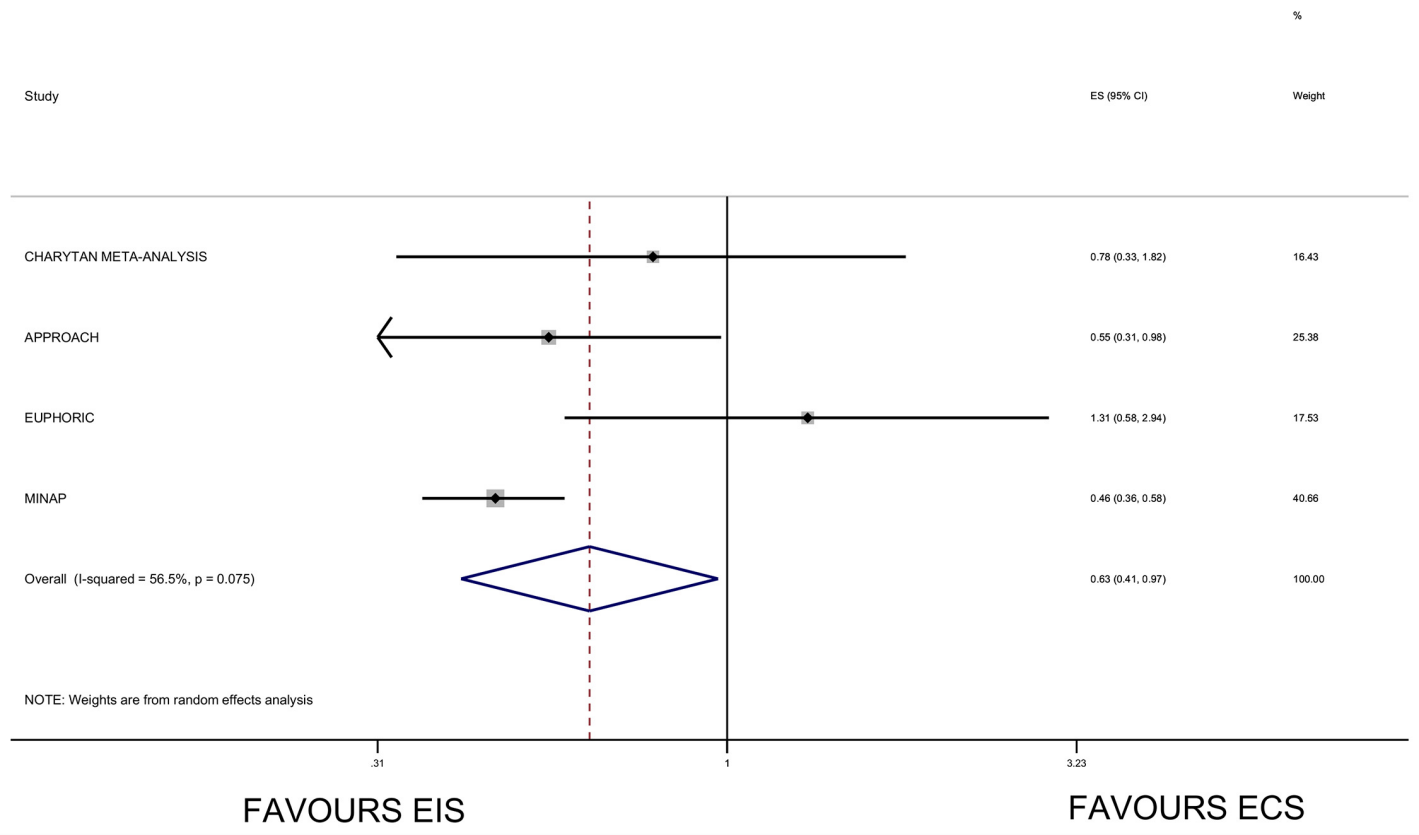
Forest plot and meta-analysis of studies reporting results in those with an eGFR<30ml/minute/1.73m^2^ for the association between an early invasive strategy and mortality compared with a conservative management strategy with results stratified by estimated glomerular filtration rate. Results from the APPROACH study were reported overall for eGFR<60ml/minute/1.73m^2^ and also stratified by eGFR category (p-interaction by eGFR 0.624). Results from the EUPHORIC study were reported overall for eGFR<60ml/minute/1.73m^2^ and also stratified by eGFR category (p-interaction by eGFR 0.31). Results from the MINAP study were reported stratified by eGFR category (p-interaction by eGFR <0.001). Effect estimates for each of the studies included in the plot: ACSIS[8]- adjusted hazard ratio; CRUSADE[9]-adjusted odds ratio; APPROACH [13]-adjusted risk ratio; Charytan *et al* [6]- risk ratio; Tai (Taiwan ACS full spectrum registry) [14]-adjusted hazard ratio; CCP[10]- adjusted odds ratio; EUPHORIC [12]- adjusted odds ratio MINAP [4]–adjusted odds ratio. Abbreviations: EIS: early invasive strategy; ECS: early conservative strategy; ES: effect estimate; 95% CI: 95% confidence interval; TAIWAN ACS: TAIWAN ACS Full Spectrum Registry; ACSIS: Acute Coronary Syndromes Israeli Survey; CRUSADE: Can Rapid Risk Stratification of Unstable Angina Patients Suppress Adverse Outcomes With Early Implementation of the ACC/AHA Guidelines; CCP: Cooperative Cardiovascular Project; FRISC II Fragmin and Fast Revascularization during Instability in Coronary Artery Disease; VINO: Value of first day angiography/ angioplasty In evolving Non-ST segment elevation myocardial infarction; TIMI IIIB: Thrombolysis in Myocardial Infarction (TIMI) IIIB clinical trial; TACTICS TIMI18: The Treat Angina with Aggrastat and determine Cost of Therapy with an Invasive or Conservative Strategy; ICTUS: Invasive versus Conservative Treatment in Unstable Coronary Syndromes; EUPHORIC: European Public Health Outcome Research and Indicators Collection Project; APPROACH: Alberta Provincial Project for Outcome Assessment in Coronary Heart Disease; MINAP: Myocardial Ischaemia National Audit Project.

Meta-analysis restricted to studies which reported data on those with CKD stage 4 or more (eGFR<30ml/minute) suggested a trend to improves survival with an early invasive strategy (RR0.63 95% CI 0.41–0.97) (Fig 4). Data were not meta-regressed due to a limited number of studies contributing to this analysis.

## Discussion

Our comprehensive search strategy identified ten studies that reported on the association between an early invasive strategy after NSTE-ACS and mortality compared with a conservative strategy in patients with CKD. Despite qualitative heterogeneity between the studies, all of the cohort studies except one unadjusted analysis reported a survival benefit associated with an early invasive approach. The meta-analysis of RCT data reported a trend to an improved survival, although the interpretation is cautiously applied as the confidence interval crossed one. Our meta-analysis of observational data also supported an early invasive approach in those with CKD. However whether the survival benefit is maintained in severe renal impairment (CKD 4 and 5) seems less clear with small sample sizes having limited the power in several of the studies. There is a lack of data addressing this important clinical question specifically in the dialysis population.

To the best of our knowledge this is the first systematic review addressing this research question to incorporate both the available observational evidence from relevant cohort studies and previous meta-analysis of RCTs. We used a comprehensive search strategy, with broad definitions of kidney disease and interventional strategy after NSTE-ACS for a thorough and inclusive search. We provide a detailed narrative summary with focus on the methodologies of the included studies. The studies identified represented a large international CKD population with NSTE-ACS and with the inclusion of several national registry based analyses reflect actual clinical practice patterns and outcomes in contemporary populations with NSTE-ACS and CKD.

Meta-analysis of the observational studies demonstrated a large degree of heterogeneity. Some of this was driven by study size. Additionally, a large proportion of this was driven by the MINAP based analysis. This study included one of the largest populations, in particular including individuals with severe CKD and a high degree of co-morbidity. It was the only study which reported in its primary analysis evidence of interaction by stage of CKD on the association between an early invasive approach and mortality. Additionally, observational studies have recognised limitations in their robustness in evaluating the comparative effectiveness of interventions [20] and cannot be interpreted as causal. Nevertheless, for the majority of data, despite the heterogeneity across definitions of various kidney function levels, it is reassuring that the qualitative picture generally supports that an early invasive approach is favoured when looking at the RCT derived and observational data jointly.

Definitions of early invasive therapy and corresponding comparison group, in particular in the cohort studies, varied. This reflects the strengths and limitations of utilising existing routinely collected data sources for research. Often these resources are designed for a different primary use- for example audit, rather than the research question being addressed. Despite the large populations included and wide range of information collected, there may be lack of granularity of information available. Bias can also be introduced. For example, studies comparing inpatient angiography with no inpatient angiography are at particular risk of immortal time bias which could result in an overestimate of the survival advantage associated with an early invasive therapy [4, 10, 12, 21]. The MINAP based report used a sensitivity analyses excluding people who died early to help evaluate the possible impact of such bias; reassuringly the findings were similar to the main analyses [4, 21]. Careful definition of the cohort so as not to advantage one group over the other is required. The APPROACH study excluded those who died within the first two days and then examined the remaining cohort, comparing those who had coronary angiography within the first two days with those that did not thus removing the risk of immortal person-time [13].

Other biases may also be important. All nine of the studies that used biochemistry to define CKD utilised a single measure of renal function to estimate CKD stage with the risk of misclassification of CKD stage. Several studies specified that the admission measure was utilised. Without pre-morbid renal function estimates this could result in cases of AKI being categorised as CKD resulting in an over-estimation of the prevalence of CKD. However, in clinical practice pre-morbid renal function may not be available with clinical decisions being made on the basis of single measures and it is likely the results reflect actual clinical practice. Linkage of registries to laboratory systems or other repositories of longitudinal clinical data may facilitate future research to understand the relative contributions of CKD and AKI on outcomes [13, 22].

In addition to all-cause mortality, one study reported the risk of AKI and progression to end stage renal disease (requiring renal replacement therapy[RRT]) associated with early invasive therapy. In the APPROACH study people who received early invasive management were modestly more likely to develop AKI but the risks of requiring dialysis and long term risk of end stage renal disease were similar, and patients had better long term survival than those treated conservatively [13]. These findings were consistent across varying levels of baseline kidney function, suggesting similar relative risks and benefits of early invasive management in people with and without pre-existing kidney disease [13]. Although lacking information on AKI/RRT the MINAP study results also support a survival benefit across the CKD stages however the statistical homogeneity of effects for early invasive therapy on survival was not observed [4]. This may reflect differences in the adjustment for confounders between the two studies, or differences in the included populations. The less conservative effect estimates observed in the MINAP study and a more parsimonious approach to the propensity score suggest that adjustment for confounding variables may not have been as effective. Statistical tests of interaction tend to be of low power and this is another possible explanation. The EUPHORIC study and meta-analysis by Charytan *et al* reported contrasting effect estimates in association with an early invasive therapy in those with an eGFR<30ml/minute/1.73m^2^ but with wide confidence intervals [6, 12]. Both studies were potentially limited in their stratified analysis by low patient numbers in this category. Importantly none of the studies reported outcomes specifically in an ESRF requiring RRT population.

There are limitations to the review. The strategy to utilise a checking process of 10% during study selection was pragmatic; reassuringly the degree of agreement was high. Data extraction and evaluation of risk of bias were undertaken by a single person. Steps including piloted data extraction forms and double checking of data were taken to reduce the potential of error being introduced. There were five studies which were unavailable in full text/English language [23–27]. A single abstract of a meta-analysis reporting outcomes after early invasive therapy in dialysis patients was identified. The data were provisionally supportive of an early invasive approach however no judgement on the quality of the studies utilised could be made-therefore the data was not included [27]. The other four abstracts did not suggest information pertaining to CKD was reported but there is a small risk that some relevant data may have been missed.

Specific data on important outcomes including recurrent myocardial infarction, stent thrombosis or length of stay were not available. These should be included in future studies.

The evidence from the observational studies in this systematic review generally support that an early invasive therapy in NSTE-ACS or UA is associated with a survival advantage in patients with early and moderate CKD. This review adds further support to the current recommendations within clinical guidance [1, 28]. However, there remains a paucity of data relating to patients with severe CKD and in particular those managed with dialysis or renal transplantation. Whether an early invasive approach is the preferred approach in these groups remains less clear. Renal and cardiology communities must work collaboratively on an inclusive approach to future trials in NSTE-ACS to understand the optimal therapeutic strategies and improve the outcomes of these high risk, under-studied patient groups.

## Supporting Information

S1 Appendix Search strategies used to identify potential studies. (DOCX)

S2 Appendix Risk of bias assessment for each of the included cohort studies (https://bmg.cochrane.org/research-projectscochrane-risk-bias-tool). (DOCX)

S3 Appendix PRISMA Checklist. (DOC)

S4 Appendix Summary of statistical methods used in the included studies and co-variables included. (DOCX)
