# Supplementary material for: Impact of an Early Invasive Strategy versus Conservative Strategy for Unstable Angina and Non-ST Elevation Acute Coronary Syndrome in Patients with Chronic Kidney Disease: A Systematic Review
Source: PLoS One. 2016 May 19;11(5):e0153478. doi: 10.1371/journal.pone.0153478 (PMC4873245; doi:10.1371/journal.pone.0153478)
Supplement: S1 Appendix — (DOCX) [file pone.0153478.s001.docx]

S1 Appendix. Search strategies used to identify potential studies

**MEDLINE search strategy**

1. explode ’Myocardial-Infarction

2. explode ’Angina-Unstable’

3. explode ’Myocardial-Infarction

4. explode ’Angina-Unstable’

5. unstable angina$

6. coronary syndrome:

7. myocardial infarct:

8. myocardial infarction heart infarct:

9. NSTE-ACS

10. unstable coronary

11. 3 or 4 or 5 or 6 or 7 or 8 or 9 or 10

12. ischaemi: adj3 guid:

13. ischemi: adj3 guid:

14. early adj3 invasive

15. invasive adj3 conservative

16. ischemi: adj3 strateg:

17. ischaemi: adj3 strateg:

18. conservative adj3 strateg:

19. conservative adj3 therap:

20. conservative adj3 treatment:

21. conservative adj3 management

22. interventional adj3 strateg:

23. interventional adj3 therap:

24. interventional adj3 treatment:

25. interventional adj3 management

26. invasive adj3 strateg:

27. invasive adj3 therap:

28. invasive adj3 treatment:

29. invasive adj3 management

30. triage adj3 angiograph:

31. 12 or 13 or 14 or 15 or 16 or 17 or 18 or 19 or 20 or 21 or 22 or 23 or 24 or 25 or 26 or 27 or 28 or 29 or 30

32. 11 and 31

**EMBASE search strategy**

1. exp heart infarction/

2. exp unstable angina pectoris/

3. Acute Coronary Syndrome/

4. unstable angina$.tw.

5. coronary syndrome$.tw.

6. myocardial infarct$.tw.

7. heart infarct$.tw.

8. NSTE-ACS.tw.

9. unstable coronary.tw.

10. or/1-8

11. (isch?emi$ adj3 guid$).tw.

12. (early adj3 invasive$).tw.

13. (early adj3 conservative$).tw.

14. (isch?emi$ adj3 strateg$).tw.

15. (conservative adj3 (strateg$ or therapy or therapies or treatment$ or management)).tw.

16. (interventional adj3 (strateg$ or therapy or therapies or treatment$ or management)).tw.

17. (invasive adj3 (strateg$ or therap$ or treatment$ or management)).tw.

18. (triage adj3 angiograph$).tw.

19. or/11-18

20. 10 and 1
