# Supplementary material for: Impact of an Early Invasive Strategy versus Conservative Strategy for Unstable Angina and Non-ST Elevation Acute Coronary Syndrome in Patients with Chronic Kidney Disease: A Systematic Review
Source: PLoS One. 2016 May 19;11(5):e0153478. doi: 10.1371/journal.pone.0153478 (PMC4873245; doi:10.1371/journal.pone.0153478)
Supplement: S2 Appendix — (DOCX) [file pone.0153478.s002.docx]

S2 Appendix. Risk of bias assessment for each of the included cohort studies (https://bmg.cochrane.org/research-projectscochrane-risk-bias-tool)

|  | **Altahan**  **(ACSIS)** | **Bhatt**  **(CRUSADE)** | **Chertow**  **(CCP)** | **Chu**  **(NHIRD)** | **Goldenberg**  **(EUPHORIA)** | **James**  **(APPROACH/**  **AKDN)** | **Lin**  **(Taiwan ACS Full Spectrum** | **Shaw**  **(MINAP)** | **Wong**  **(ACS I, ACS II)** |
| --- | --- | --- | --- | --- | --- | --- | --- | --- | --- |
| **1. Was selection of exposed and non‐exposed cohorts drawn from the same population?** | Yes: low risk | Yes: low risk | Yes: low risk | Yes: low risk | Yes: low risk | Yes: low risk | Yes: low risk | Yes: low risk | Yes: low risk |
| **2. Can we be confident in the assessment of exposure?** | Data were collected on pre-specified forms; no mention of validation.  Medium risk | Pre-specified standardised forms; no mention of validation.  Medium risk | Trained nurses and medical records technicians abstracted data from hospital records and data were entered directly into a computerised database management system; no comment re validation of data.  Medium risk | Data obtained from the national register used for reimbursement claims; no comment on validation is provided but it is likely that claims for procedures is accurate. Medium/low risk | Similar case report forms and the same definitions for ACS.  Medium risk | Data were collected prospectively using standardised definitions from established regional registry; No specific mention of validation procedures.  Medium/low risk | Standardised case report forms were utilised with validation check in 5% of all forms at each recruiting site undertaken. Medium/low risk | Data were collected prospectively using standardised definitions. Brief mention of data validation and quality assurance procedures are provided.  Medium/low risk | Data were recorded on standardised reporting forms by local study co-ordinator or physician.  Standardised definitions; central data checks with queries checked with the participating centres. Medium/low risk |
| **3. Can we be confident that the outcome of interest was not present at start of study** | Yes-low risk | Yes-low risk | Yes-low risk | Yes-low risk | Yes-low risk | Yes-low risk | Yes-low risk | Yes- low risk | Yes-low risk |
| **4. Did the study match exposed and unexposed for all variables that are associated with the outcome of interest or did the statistical analysis adjust for these prognostic variables?** | Some adjustment and included a propensity score approach.  Medium risk | Some adjustment.  Medium risk | Some adjustment.  Medium risk | Univariable analysis only. High risk | Some adjustment.  Medium risk | Extensive inclusion of co-variables. 1-1 matching on propensity score.  Medium/low risk | Some adjustment.  Medium risk | Some adjustment.  Medium risk | Univariable analysis only. High risk |
| **5. Can we be confident in the assessment of the presence or absence of prognostic factors?** | As per question 2.  Medium risk | As per question 2.  Medium risk | As per question 2.  Medium risk | As per question 2. ICD9 codes used to identify co-morbidity and outcomes. No information given as to the validity. Medium risk | As per question 2.  Medium risk | Data also obtained from administrative health care sources and laboratory data.  Medium/low risk | As per question 2.  Medium/low risk | As per question 2.  Medium/low risk | As per question 2.  Medium risk |
| **6. Can we be confident in the assessment of outcome?** | Outcome mortality.  Mortality data ascertained from hospital charts and the Israeli National Population Registry.  Low risk | Outcome mortality.  Pre-specified standardised forms and data collected during hospital admission.  Low risk | Outcome mortality.  No specific mention of how outcome ascertained in this paper. From a previous CCP publication data was obtained from the Medicare Enrolment database (1).  Low risk | Outcome mortality.  As per question 5.  Low risk | Outcome mortality.  In patient case follow-up within each of the registries  Low risk | Outcome mortality.  Mortality data from provincial vital statistics records  Low risk | Outcome mortality.  Patients were followed up at 3,6,9 and 12 months. No specific mention on how this was undertaken in this paper, or in the original description of the registry(2).  Low/Medium risk | Outcome mortality.  Mortality data from NHS central register via linkage obtained.  Low risk | Outcome mortality.  ACS 1 and II 1 year outcomes ascertained by telephone interview  (F/U data not available for N=390, 7.9%).  Low/Medium risk |
| 7. Was the follow up of cohorts adequate? | No missing outcome data reported.  Low risk | No missing outcome data reported.  Low risk | No missing outcome data reported.  Low risk. | No missing outcome data reported.  Low risk. | No missing outcome data reported.  Low risk. | No missing outcome data reported.  Low risk. | No missing outcome data reported.  Low risk. | Details of missing data and sensitivity analyses using multiple imputation included. Follow-up complete on the complete case cohort.  Low risk | F/U data not available for N=390, 7.9%). No description as to whether  excluded patients varied in terms of exposure or outcome in this manuscript  Medium risk |
| **8. Were co‐Interventions similar between groups?** | Medication data only provided by kidney function category; individuals with kidney disease were less likely to be prescribed range of medications including aspirin, heparin, clopidogrel, beta-blockers**;**  These treatments were not included in the analysis;  Medium/high risk | Individuals in the early invasive group were more likely to be prescribed various medications including aspirin, clopidogrel, beta-blockers, heparin, G2b,3a inhibitors. These treatments were not included in the analysis;  Medium /high risk | Data not provided; High risk | Patients with CKD and managed with EIS were more likely to be prescribed various medications including aspirin, beta-blockers, ACEi than those with CKD managed with ECS. These treatments were not included in the analysis.  Medium/high risk | Medication data only provided by kidney function category; individuals with kidney disease were less likely to be prescribed ACE inhibitors, aspirin, beta-blockers, statins.  These treatments were included in the analysis;  Medium risk | Medication data provided for the propensity matched cohort overall (ie not stratified by eGFR)  Patients undergoing EIA were more likely to be prescribed aspirin, thienopyridines, b-blockers, ACEi/ARB, statin. Medications were not included in the survival analysis model  Medium risk | Medication data only provided by kidney function category and for the combined STE-and NSTE- populations. Individuals with kidney disease were less likely to be prescribed ACE inhibitors, clopidogrel, beta-blockers, and statins.  Medications were included in the survival analysis regression model.  Medium risk | Data not provided although sensitivity analyses including medications provided.  Medium/high risk | Medication data only provided by kidney function category; individuals with kidney disease were less likely to be prescribed aspirin, thienopyridines, heparin or G2b/3a inhibitors.  High risk |
| **Overall risk of bias: high, average, low** | Medium | Medium | Medium | Medium/high | Medium | Medium/low | Medium | Medium | Medium/high |

Abbreviations: ACS: acute coronary syndrome; CKD: chronic kidney disease; ACE i:angiotensin converting enzyme inhibitor; ECS: early conservative strategy; EIS: early invasive strategy; F/U: follow-up; eGFR: estimated glomerular filtration rate (ml/minute/1.73m^2^);
