# Supplementary material for: Impact of an Early Invasive Strategy versus Conservative Strategy for Unstable Angina and Non-ST Elevation Acute Coronary Syndrome in Patients with Chronic Kidney Disease: A Systematic Review
Source: PLoS One. 2016 May 19;11(5):e0153478. doi: 10.1371/journal.pone.0153478 (PMC4873245; doi:10.1371/journal.pone.0153478)
Supplement: S4 Appendix — (DOCX) [file pone.0153478.s004.docx]

S4 Appendix. Summary of statistical methods used in the included studies and co-variables included

| **Main author**  **(data source)** | **Altahan**  **(ACSIS)** | **Bhatt**  **(CRUSADE)** | **Chertow**  **(CCP)** | **Chu**  **(NHIRD)** | **Goldenberg**  **(EUPHORIA)** | **James (APPROACH/**  **AKDN)** | **Lin (Taiwan**  **ACS Full Spectrum)** | **Shaw**  **(MINAP)** | **Wong**  **(ACS I, ACS II)** |
| --- | --- | --- | --- | --- | --- | --- | --- | --- | --- |
| Main statistical methods | Cox regression adjusted for propensity to undergo EIA | GEE logistic regression models;  Secondary analysis with results stratified by renal function | Multivariable logistic regression model | Kaplan Meier survival curves | GEE (hospital) logistic regression with interaction by eGFR | - 1. propensity score matching;   Stratified Cox regression with interaction term by eGFR | Multivariable Cox regression model | Multivariable logistic regression model with interaction term by eGFR and robust SE; propensity score based sensitivity analysis | Proportion of patients in the EIS and ECS groups who died within 1 year stratified by eGFR category and |
| **Co-variables included in final model** | | | | | | | | | |
| Socio-demographic | age, sex, , current smoking status | age, sex, BMI , ethnicity, Fhx CAD; insurance status, hospital characteristic ,hours of presentation | age, sex, ethnicity, state of residence, hospital size, hospital local (rural/non rural), teaching hospital |  | age, sex, current smoking status | Age, sex, smoking status, Fhx, hospital | Age, sex, smoking status, Fhx | age, ethnicity, sex, IMD score, current smoking status and hospital | . |
| Co-morbidity | DM, HTN, MI, CHF, | HTN, DM smoking status, hypercholesterolemia, MI, PCI, CABG, CHF, CVSD, renal disease | cancer, DM, PVD, COPD,MI, Stroke, |  | DM,HTN, CAD | HTN, DM, hyperlipidaemia, previous MI, previous PCI, previous CABG, CHF, PVD, CVSD, Charlson co-morbidity score | Dyslipidaemia, HTN, DM | history of angina, hyperlipidaemia, HTN, PVD, CVSD, COPD, CHF, previous PCI, previous CABG, previous MI, DM, |  |
| Physiological derangement | Killip class=/>II | ST-segment depression, transient ST segment elevation, positive cardiac markers, signs of CCF, heart rate, SBP | conduction disturbances, heart rate, serum albumin |  | Killip class>/= II; presence of KD, | Albuminuria, anaemia,TIMI score, elevated cardiac markers (CK, Trop T or I), hypotension, tachycardia, ischaemia on ECG |  | SBP, heart rate, Hb,peak troponin, ECG  changes |  |

Abbreviations: EIA: early invasive therapy; GEE: generalised estimating equations; eGFR: estimated glomerular filtration rate; CrCl: Creatinine clearance; BMI: body mass index;

Fhx: family history; DM: diabetes, HTN: hypertension; MI: myocardial infarction; CCF: congestive cardiac failure; PVD: peripheral vascular disease; COPD: chronic obstructive pulmonary disease; CAD: cardiovascular disease;TnT: troponin T; IL6: interleukin; SBP: systolic blood pressure; TIMI: Thrombolysis in myocardial infarction score

1. Marciniak TA, Ellerbeck EF, Radford MJ, et al.: Improving the quality of care for medicare patients with acute myocardial infarction: Results from the cooperative cardiovascular project. *JAMA,* 279**:** 1351-1357, 1998

2. Shyu K, Wu C, Mar G, Hou Charles J, Li A, Wen M, Lai W, Lin S, Kuo C, Hwang J, Chiang F: Clinical characteristics, management and in-hospital outcomes of patients with acute coronary syndrome - observations from the Taiwan ACS full spectrum registry. *Acta Cardiol Sin,* 27**:** 135 - 144, 2011
